# Monitoring of Nesting Songbirds Detects Established Population of Blacklegged Ticks and Associated Lyme Disease Endemic Area in Canada

**DOI:** 10.3390/healthcare8010059

**Published:** 2020-03-13

**Authors:** John D. Scott, Emily L. Pascoe, Muhammad S. Sajid, Janet E. Foley

**Affiliations:** 1 International Lyme and Associated Diseases Society, 2 Wisconsin Circle, Suite 700, Chevy Chase, MD 20815-7007, USA; 2 School of Veterinary Medicine, Department of Medicine and Epidemiology, University of California Davis, One Shields Avenue, Davis, CA 95616-8734, USA; elpascoe@ucdavis.edu (E.L.P.); mssajid@ucdavis.edu (M.S.S.); jefoley@ucdavis.edu (J.E.F.)

**Keywords:** songbirds, nesting, fledgling, ticks, established population, *Ixodes scapularis*, *Haemaphysalis leporispalustris*, Canada

## Abstract

This study provides a novel method of documenting established populations of bird-feeding ticks. Single populations of the blacklegged tick, *Ixodes scapularis*, and the rabbit tick, *Haemaphysalis leporispalustris*, were revealed in southwestern Québec, Canada. Blacklegged tick nymphs and, similarly, larval and nymphal rabbit ticks were tested for the Lyme disease bacterium, *Borrelia burgdorferi* sensu lato (Bbsl), using PCR and the flagellin (*flaB*) gene, and 14 (42%) of 33 of blacklegged tick nymphs tested were positive. In contrast, larval and nymphal *H. leporsipalustris* ticks were negative for Bbsl. The occurrence of Bbsl in *I. scapularis* nymphs brings to light the presence of a Lyme disease endemic area at this songbird nesting locality. Because our findings denote that this area is a Lyme disease endemic area, and *I. scapularis* is a human-biting tick, local residents and outdoor workers must take preventive measures to avoid tick bites. Furthermore, local healthcare practitioners must include Lyme disease in their differential diagnosis.

## 1. Introduction

Blacklegged ticks, *Ixodes scapularis* (Acari: Ixodidae), have a native range east of the Rocky Mountains, and are vectors of multiple enzootic pathogens. This human-biting ectoparasite is known to harbor and transmit at least nine different tick-borne, zoonotic pathogens [1]. Songbirds (Order: Passeriformes) are well known to widely disperse *I. scapularis* larvae and nymphs in Canada extending as far west and as far north as northern Alberta [2,3,4,5,6,7,8]. During northbound migratory flights, certain avifauna have transcontinental or trans-equatorial flights [9,10]. These long-distance passerines fly hundreds of kilometers, and transport *Amblyomma* ticks, including *A. americanum* [2], *A. dissimile* [11], *A. humerale* [12], *A. imitator* [4], *A. maculatum* [2,3], *A. rotundatum* [13], *A. sabanerae* [14], and *Ixodes* ticks, namely *I. affinis* [15], *I. minor* [16], *I. scapularis* [2,4,5,6,7,8] into Canada from the southern United States and the Neotropics. During the nesting and fledgling period, passerines make multiple flights back and forth to the nest. When these ground-foraging songbirds are parasitized by a number of co-infesting blacklegged ticks, they have the potential to initiate new geographical foci of ticks in outlying areas [17,18,19].

Most established populations of *I. scapularis* are infected with the Lyme disease bacterium, *Borrelia burgdorferi* sensu lato (Bbsl) [20]. Worldwide, the Bbsl complex consists of at least 24 genospecies (*Borrelia maritima* being the latest entry); the most common borrelial genospecies in the Temperate Zone of North America is *Borrelia burgdorferi* sensu stricto (Bbss), which is pathogenic to humans and several domestic animals [21]. At least six Bbsl genospecies in North America are known to be pathogenic to humans. Epidemiologically, at least seven human-biting *Ixodes* species act as vectors of Lyme disease spirochetes in Canada, namely *Ixodes angustus*, *Ixodes cookei* (groundhog tick), *Ixodes muris* (mouse tick), *Ixodes pacificus* (western blacklegged tick), *Ixodes scapularis* (blacklegged tick), and *Ixodes spinipalpis* [8,22,23,24].

When the tick salivary glands are infected with Lyme disease spirochetes, *Ixodes* ticks can transmit Bbsl to the host in less than a day [25], especially during nymphal blood meals. Based on North American studies, Bbsl is normally transmitted in 24–48 h; however, when the salivary glands are infected with Lyme disease spirochetes, transmission to a host can occur in less than 16 h [26,27]. Using European Bbsl strains and *Ixodes ricinus* ticks, Bbsl has been found disseminated in mice within 12 h [26].

In order for a location to meet the criteria for an established population of *I. scapularis*, an area must have at least six individual ticks or at least two of the three motile life stages identified within a single collection period [28]. Normally the collection period is one year; however, in the present study, it was two months.

The aim of this study was: (1) to determine whether tick-infested songbirds monitored during the nesting period are a means of pinpointing unrecognized populations of ticks and (2) to ascertain if these bird-feeding ticks are infected with tick-borne, zoonotic pathogens.

## 2. Materials and Methods

### 2.1. Tick Collection

**Figure 1:**
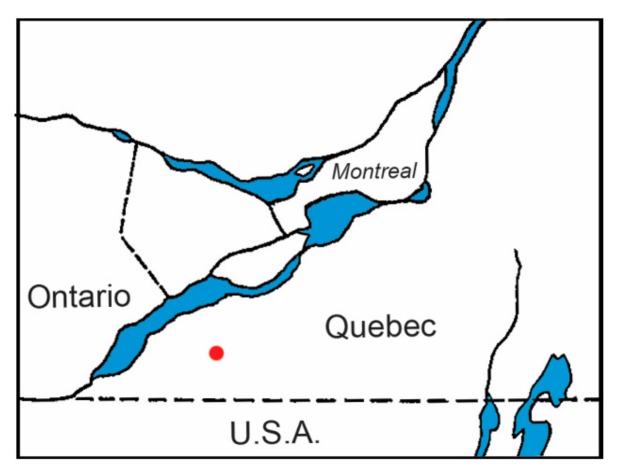
Map shows the songbird monitoring site at Montée Biggar, Québec. The red dot indicates the location of an established population of blacklegged ticks and associated Lyme disease endemic area.

Ticks were collected from songbirds during the period 8 June to 2 August 2019 at Montée Biggar (45.088226° N, 74.215678° W) in southwestern Québec, Canada (Figure 1). Live ticks were collected from passerines using hardened stainless steel, #5, superfine-tipped forceps (BioQuip Products, Rancho Dominguez, CA). Live ticks were put into a transparent 8.5 mL, 15.7 mm × 75 mm, round-bottomed polypropylene tube (Sarstedt, Montréal, Québec, CA); each tube contained ticks from a single host. In order to keep live ticks from escaping, a piece of tulle netting (3-cm diameter) was placed over the mouth of the tube, and a push cap, which had a 7-mm hole, was promptly inserted into tube opening. This technique prevented live ticks from escaping and, at the same time, provided ventilation for live ticks. For mailing, polypropylene tubes were put in a double-zipper plastic bag with slightly moistened paper towel to sustain high humidity. All ticks were sent directly to the lab (J.D.S.) for identification. Live, partially and fully engorged larval and nymphal ticks were held to molt (metamorphose to the next developmental life stage) to confirm the species identification and, in certain cases, reveal transstadial passage of tick-borne, zoonotic pathogens.

Taxonomic keys and scientific articles were employed to confirm ixodid tick identifications, namely *I. scapularis* larvae [29,30] and nymphs [30,31] and, likewise, *Haemaphysalis leporispalustris* (rabbit tick) larvae [29] and nymphs [32].

Engorged ticks were exposed to a long-day photoperiod of 16:8 (L:D) h. Background and processing information (i.e., host species, leg band number, geographic location, date collected, collector's name, developmental life stage, degree of engorgement) were logged for ticks from each avian host. After identification, ticks were stored in 2-mL microtubes (Sarstedt, Montréal, Québec, Canada) containing 95% ethyl alcohol to preserve them and stabilize DNA.

### 2.2. Spirochete Detection

DNA was extracted from ixodid ticks preserved in 95% ethyl alcohol using the Qiagen DNeasy Blood and Tissue Kit (Qiagen, Valencia, CA, USA) following the manufacturer's instructions. Quantitative real-time PCR (qPCR) was used in a combined thermocycler/fluorometer (ABI Prism 7700; Applied Biosystems, Foster City, CA) to screen for *B. burgdorferi* s.l. DNA. A probe (6FAM-TTC-GGT-ACT-AAC-TTT-TAG-TTA-A) with a 3’ end modified with minor groove binding protein and labelled with corresponding dye, forward (5’-GCT-GTA-AAC-GAT-GCA-CAC-TTG-GT-3’) and reverse (5’-GGC-GGC-ACA-CTT-AAC-ACG-TTA-G-3’) primers targeting a region of the 16S rRNA gene were used [33,34]. Water negative controls were included in each run, while positive controls consisted of DNA from cultured Bbsl. Ticks were considered positive if the cycle threshold was <40, and there was a characteristic amplification curve. To identify *B. burgdorferi* s.l. genospecies in qPCR-positive samples, conventional PCR using primers (5’-AAR-GAA-TTG-GCA-GTT-CAA-TC-3’ and 5’-GCA-TTT-TCW-ATT-TTA-GCA-AGT-GAT-G-3’) that target the flagellin (*flaB*) gene were performed as previously described [35]. Modifications were made to use GoTaq Green Master Mix (Promega, Madison, WI). Gel electrophoresis was performed on conventional PCR products using 1% agarose gel stained with Gelstar (Lonza, Rockland, ME). Amplicons of approximately 497 bases were excised from the gel and purified using the QIAquick Gel Extraction Kit (Qiagen, Valencia, USA), and sequenced using an ABI 3730 sequencer (Davis Sequencing, Davis, CA). Results were examined for accuracy of base determination, and end-read errors were trimmed [36]. We compared sequences to those in the GenBank database using the Basic Local Alignment Search Tool (BLAST), National Center for Biotechnology Information (NCBI), Bethesda, MD [37]. The MUSCLE algorithm was used to perform sequence alignments for *fla* gene sequences from ticks and from reference *Borrelia* genospecies obtained from the NCBI GenBank database [38].

## 3. Results

### 3.1. Tick Collection

In total, 68 ticks were collected from 23 songbirds representing five families within the Order Passeriformes (Table 1). These songbird-derived ticks include 64 blacklegged ticks (nymphs, *n* = 63; larva, *n* = 1) and four *H. leporispalustris* (nymphs, *n* = 3, larva, *n* = 1). It is noteworthy that all tick collections were made before southward migration started and, therefore, all ticks were locally acquired.

In one particular bird parasitism, a Veery was parasitized by three engorged *I. scapularis* nymphs, and one fully engorged nymph molted to a male in 31 d. As well, another Veery was parasitized by two engorged *I. scapularis* nymphs, and a partially engorged nymph molted to a male in 33 d. In addition, a partially engorged *I. scapularis* nymph was collected from a Rose-breasted Grosbeak, and it molted to a female in 40 d; this female was positive for Bbsl. In the present study, Veeries were the most frequently occurring songbirds followed by Common Yellowthroats (Table 1).

### 3.2. Spirochete Detection

All ticks testing positive for Bbsl belonged to the genospecies *B. burgdorferi* sensu stricto. Significantly, 14 (42%) of 33 *I. scapularis* nymphs tested were positive (Table 1). A single *I. scapularis* larva was collected, but not tested for Bbsl. All larval and nymphal *H. leporispalustris* were negative for Bbsl. Four *I. scapularis* nymphs were collected from an American Robin on 22 July 2019, and each was positive for Bbsl. Of epidemiological merit, five (100%) of five *I. scapularis* nymphs collected from American Robins were positive for Bbsl. As well, seven *I. scapularis* nymphs collected from an American Robin were pooled for Bbsl testing; the pool was positive for Bbsl. This pool was not included in the Bbsl prevalence calculations. A total of 23 ticks were not tested for Bbsl because they got hot in transit, and died and spoiled.

Bbsl DNA sequences from this study were submitted to GenBank, and the accession numbers are listed in Table 2.

## 4. Discussion

In this study, we exposed *I. scapularis* and *H. leporispalustris* populations by monitoring a wooded area in southwestern Québec during the nesting and fledgling period. This deciduous woodland turned out to be a breeding colony of *I. scapularis* infected with Lyme disease spirochetes. The presence of Bbsl in these bird-feeding *I. scapularis* nymphs indicates that this nesting area is endemic for Lyme disease. Most importantly, these bird parasitisms during the nesting and fledgling periods provide a unique means of identifying established populations of ticks.

### 4.1. Nesting Area Marks Established Population of Blacklegged Ticks

**Figure 2:**
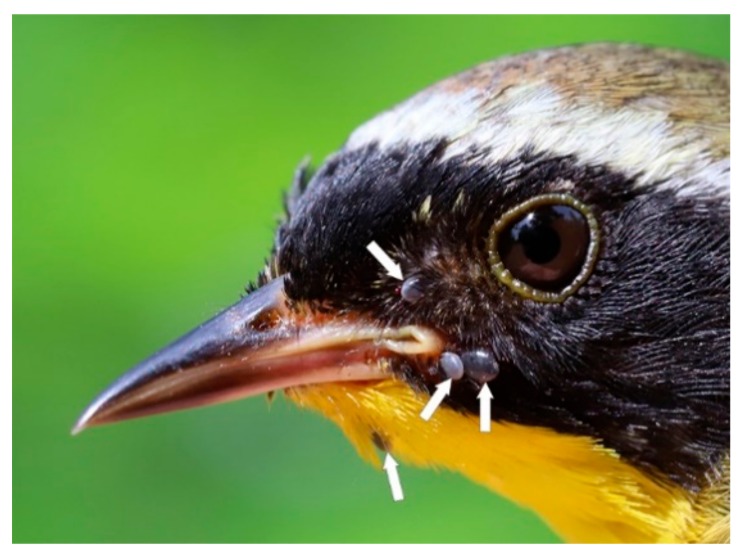
Common Yellowthroat, after-second year male, infested with 12 *I. scapularis* nymphs (eight not visible). This multi-tick bird parasitism brings to light the potential of this bird, and the attached nymphs, to establish a new population of blacklegged ticks. Photo credits: Ana Morales.

Firstly, a Common Yellowthroat parasitized by 12 *I. scapularis* nymphs signifies an established population in this nesting environs (Figure 2) [28]. Secondly, a Veery was parasitized by immature stages of *I. scapularis* (two nymphs, one larva), and these two developmental stages on a single host within a single year, indicate that this locale is an established population [28]. When engorged *I. scapularis* nymphs molt, they become adults in late June to mid-August. Consistent with other bird-tick-Bbsl studies reporting Bbsl infection prevalence which range from 31% to 59% in *I. scapularis* nymphs in the Great Lakes area [5,7,8,14], we obtained an infection prevalence of 42%. Since these Bbsl-infected nymphs will molt to adults, we extrapolate that the Bbsl infection prevalence for *I. scapularis* adults will be approximately 42% at this locale. Thirdly, when we combine all of the three motile life stages (larvae, nymphs, adults), there is an established population in this woodland locality. In three different ways, we meet the surveillance criteria for an established population of *I. scapularis* ticks [28]. Based on the Bbsl infection prevalence, we show that this area is clearly endemic for Lyme disease.

The determination of whether blacklegged ticks form an established population in an area is often determined by active surveillance. However, in this study, we have shown that ticks collected from songbirds during the nestling and fledgling period are, indeed, an established population. The June–July period is the time of year when songbirds stay in close proximity to the nest. Ecologically, the present bird-tick-pathogen study was conducted during the nesting and fledgling period, which coincides with the peak questing period of nymphal blacklegged ticks [39]. As a result, ground-foraging songbirds are frequently parasitized by host-seeking *I. scapularis* nymphs.

Cricetid rodents, such as deer mice (*Peromyscus maniculatus*) and eastern chipmunks (*Tamias striatus*), act as reservoir hosts for Lyme disease spirochetes, and maintain an enzootic transmission cycle of Bbsl, year-round [40,41,42]. In an established breeding colony of bird-feeding ticks, overwintering songbirds act as maintenance hosts, whereas migratory songbirds are incidental hosts [17]. Because migratory songbirds widely disperse ticks during bidirectional migration, heavily-infested passerine migrants can initiate Bbsl-endemic foci in new areas hundreds of kilometers away from their original source [17,18,19].

### 4.2. Short-Distance Flights Signify Tick Populations

During the nesting and fledgling period, songbirds forage contiguous to the nest, and maintain short-distance trips back and forth for food. Short runs ensure that the eggs in the nest stay warm and protected from predators. Additionally, during the fledgling period, ground-foraging songbirds make hasty flights near the nest, and promptly return to feed their young. As the ground-frequenting songbirds forage through the leaf litter or grass refuge searching for arthropods, they may become parasitized by host-seeking (questing) ticks. In the present study, bird banders, who were conducting demographic monitoring and collecting data related to songbird activity, had the opportunity to collected bird-feeding ticks. Notably, in late July, songbirds are in moult (shed and replace feathers), and only have localized flight activity. Based on the criteria for an established population [28], we were able to ascertain that there were solo populations of *I. scapularis* and *H. leporispalustris* ticks in the nesting and fledgling area at Montée Biggar, Québec. We were also able to determine that this nesting area is endemic for Lyme disease.

### 4.3. Songbirds as Reservoir Hosts

**Figure 3:**
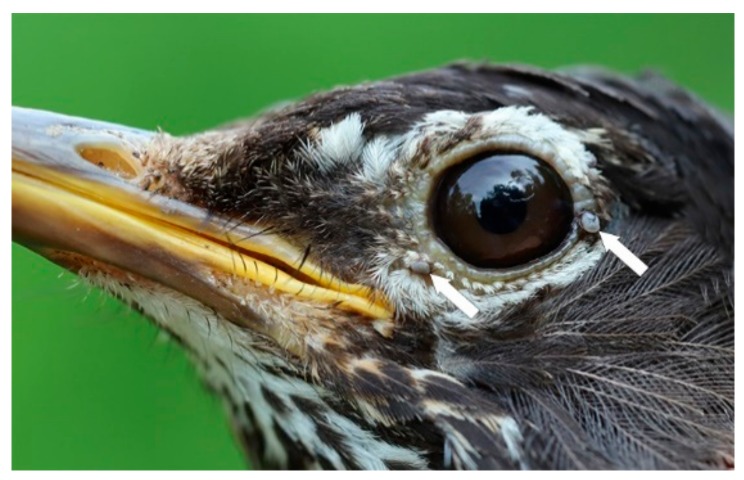
American Robin, second year female, parasitized by four *I. scapularis* nymphs (two not visible). On 22 July 2019, this bird was in moult in preparation for the upcoming, southbound, fall migration. Photo credits: Simon Duval.

Certain bird species are reservoir hosts of Bbsl. An American Robin (female, second year) was parasitized with four *I. scapularis* nymphs, and each nymph was infected with Bbsl (Figure 3). Anderson et al. isolated Lyme disease spirochetes from the liver of a Veery [43] and, also, from *I. scapularis* larvae feeding on this avian host. These acarologists also isolated Bbsl from larval *I. scapularis* feeding on Rose-breasted Grosbeaks and Common Yellowthroats suggesting that these songbirds are also reservoir-competent hosts. Other researchers provide substantive evidence that the Veery, Song Sparrow, Common Yellowthroat are competent reservoirs of Bbsl [17,44,45]. Using xenodiagnostic methods, Richter et al. placed unfed spirochete-free *I. scapularis* larvae on Bbsl-inoculated American Robins [46] and, when replete, let these fully engorged larvae molt to nymphs. These unfed nymphs were then placed on spirochete-free American Robins and, subsequently, became infected with Lyme disease spirochetes. These findings reveal that American Robins are competent reservoirs. Similarly, Anderson et al. found that American Robins are reservoir hosts of Bbsl [18]. In the present study, five (100%) of five *I. scapularis* nymphs parasitizing American Robins were infected with Bbsl. In addition, six (32%) of 19 *I. scapularis* nymphs collected from Veeries were infected with Bbsl. Since we did not take any blood samples from host birds, we do not know whether any songbirds were spirochetemic for Bbsl.

Of enzootic significance, a partially engorged *I. scapularis* nymph molted to an unfed female, and this female was positive for Bbsl; this molt demonstrates transstadial passage of Bbsl. This enzootic finding show that Bbsl can move to the next developmental life stage at this site.

### 4.4. H. leporispalustris Vector Competency

In this study, we did not identify Bbsl in *H. leporispalustris* ticks. However, Banerjee et al. detected Lyme disease spirochetes in *H. leporispalustris* ticks collected from a snowshoe hare, *Lepus americanus*, in northern Alberta [47]. As well, Scott et al. discovered *Borrelia lanei*-like spirochetes and a *Babesia divergens*-like piroplasm concurrently in a *H. leporispalustris* (female) collected from an eastern cottontail, *Sylvilagus floridanus*, in southern Manitoba [48]. Scott & Durden provide the first record of a Bbsl-positive *H. leporispalustris* (nymph) collected from an avian host (Swainson's Thrush) in Canada [14]. Previously, Scott et al. found Bbsl in a *H. leporispalustris* larva parasitizing a passerine (Canada Warbler) in Québec suggesting that this tick species is a reservoir-competent host [8]. During southbound, fall migration in Canada, larval and nymphal *H. leporispalustris* frequently parasitize passerines, and are widely dispersed in southern regions.

### 4.5. Ticks Co-infest Songbirds

**Figure 4:**
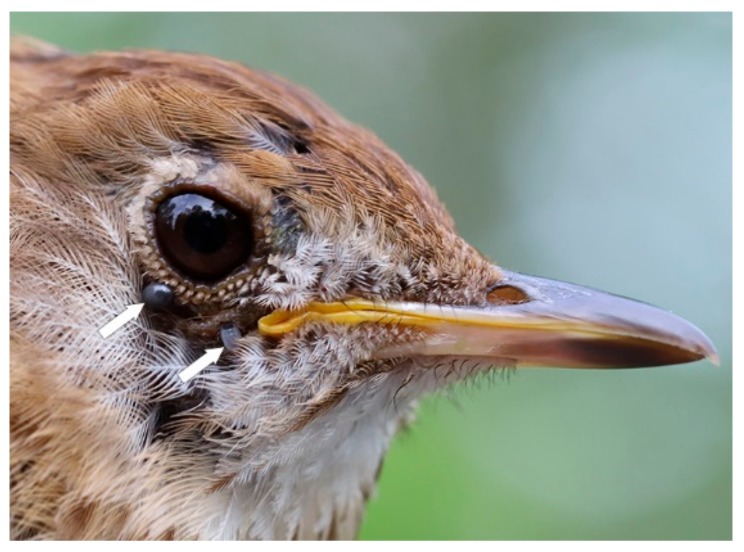
Veery, after-second year male, infested with three *I. scapularis* nymphs. One fully engorged nymph moulted to a male in 31 d. On 22 July 2019, this bird was moulting to prepare for the southward flight to its wintering range in Brazil. Photo credits: Simon Duval.

One Veery was co-infested with *H. leporispalustris* (one nymph and one larva), and *I. scapularis* (two nymphs). Not only is there a breeding colony of *I. scapularis* present in this Laurentian River basin, an established population of *H. leporispalustris* is also there. Since these ixodid ticks were collected during the nesting and fledgling period, this bird parasitism denotes a cohabitation of two tick species in this sylvan locale, and signifies that these two tick species are sympatric. The Veery has trans-border and trans-equatorial migration during its northward spring flight, and has a breeding range in southern Canada, including southwestern Québec and northern United States; the wintering range is in central and southeastern Brazil (Figure 4). During the breeding, nesting, and fledgling period, Veeries have localized activity in juxtaposition to the stationary nest. When the young have fledged the nest, these passerines replenish their fat reserves, and prepare for the southbound trek to wintering ranges in southern latitudes during August and September. In late July, they typically moult in preparation for the southbound marathon flight.

### 4.6. Definition of Lyme Disease Endemic Area

We put forth the following definition for a Lyme disease endemic area based on comprehensive, tick and Lyme disease research across Canada [8], and similarly, conducting field studies and monitoring multiple established tick populations [4,8,12,14,19,22,48,49,50,51,52,53,54,55,56,57,58]: A Lyme disease endemic area is defined as an established population of vector ticks infected with the Lyme disease bacterium, *Borrelia burgdorferi* sensu lato, at a geographic locality in a single collection period. The present bird–tick–pathogen study at Montée Biggar, Québec meets this definition for a Lyme disease endemic area.

### 4.7. Clinical Manifestations of Lyme Disease

Lyme disease is an insidious, multisystem spirochetosis that involves many body systems, including cardiac, cutaneous, endocrine, gastrointestinal, genitourinary, musculoskeletal, neurological, otologic, ophthalmological, encephalitic, and neuropsychiatric [59,60]. Only 14%–41% of Lyme disease patients remember a tick bite [61,62], and 9%–39% exhibit an erythema migrans (EM) rash; more than 50% of those with a rash have a homogenous rash [61,63,64]. Bbsl is pleopmorphic with diverse forms (i.e., granules, blebs, spherocytes, spirochetes) and, combined together in a gelatinous matrix, these forms become biofilms [65,66,67]. Lyme disease spirochetes can sequester in protected niche reservoirs, including brain [68,69,70,71], bone [72], eye [73], collagenous tissues (ligaments, tendons) [74,75], heart [71,76], kidney [71], liver [71], muscle [77], synovial cells [78], central nervous system [79,80], glial and neuronal cells [81,82,83], and scar tissue [84].

Because Lyme disease patients often experience lengthy delays in diagnosis and treatment, these patients frequently become persistent cases that result in longstanding Bbsl infections (chronic Lyme disease) [69,72,74,75,78,85,86,87,88,89,90,91,92,93,94,95,96,97,98,99,100]. Chronic Lyme disease is defined as a persistent Bbsl infection of at least six months duration [59,60,71,100]. Persistent spirochetal infection has been demonstrated in many Lyme disease patients by PCR and culture [68,71,73,74,86,87,90,91]. Whenever there is continual spirochetemia, approximately 63% of patients infected with Bbsl develop chronic Lyme disease [101]. Patients with this spirochetosis are frequently seronegative especially when serological testing falls outside the peak immune response period, typically 6 to 8 wk after initial infection. Only 48% of patients with persistent Lyme disease test positive using the two-tier serological testing system [102]; dissimilar to AIDS testing, seronegativity is common in Lyme disease patients [103]. Even though Lyme disease patients are seronegative, they may still have active infection because Bbsl side-steps the immune response [71,77,86,88,89,93,96,104,105,106]. The Lyme disease spirochete is able to evade the host immune response using a mechanism called stealth pathogenesis. When biofilm busters are implemented prior to blood draw, they help to activate the immune response and provide more reliable serological testing [107]. Psychiatric illnesses, caused by Bbsl, may include violence, substance abuse, and developmental disabilities [108,109,110]. Chronic Lyme disease frequently causes severe disability, and potentially gives rise to central nervous system complications and cognitive impairment [49,50,61,90]. Under the dire duress of chronic Lyme disease, patients resort to suicide [108,109,110], and critically ill patients ultimately have fatal outcomes caused by advanced Bbsl spirochetosis [68,71,73,88,111,112]. Bbsl may be potentially transmitted to a partner via intimate relations [89,113]. Gestational Lyme disease takes place when pregnant mothers with Lyme disease pass Bbsl spirochetes across the placenta by vertical transmission from mother to the fetus in utero [114,115,116,117,118,119].

## 5. Conclusions

By monitoring nesting songbirds, we demonstrate a novel method of pinpointing tick populations. Nesting songbirds have short-distance flight, and reflect bird-feeding ticks acquired locally. By testing songbird-derived ticks during the nesting and fledgling period, we obtained an infection prevalence of 42% for *I. scapularis* nymphs, and discovered that this locality is a Lyme disease endemic area. This high level of Bbsl infectivity, reveals that outdoor people working in this newfound, Lyme disease endemic area during snow-free days encounter a public health risk. Any individuals frequenting this area should take extra precautions to avoid tick bites, and do full-body tick checks at the end of the day. Because *Borrelia burgdorferi* sensu stricto is pathogenic to humans, anyone who is bitten by a tick or has Lyme disease symptoms should seek medical attention. Since chronic Lyme disease is a pernicious, debilitating infection, healthcare practitioners must take special steps to screen symptomatic patients for this incapacitating spirochetosis.

## Figures and Tables

**Table 1:** Detection of *Borrelia burgdorferi* sensu stricto in ticks collected from nesting songbirds captured at Montée Biggar, Québec, 8th June to 2nd August 2019.

Bird Species	No. of Hosts	No. of Ticks	No. of Ticks Pos./No. Ticks Tested				
			Hlp		Isc		*I. scapularis* Nymphs (%)
			Larva	Nymphs	Larva	Nymphs	
American Robin, *Turdus migratorius* L.	3	14 *	0/0	0/0	0/0	5/5, 2NT	5/5 (100)
Common Yellowthroat, *Geothlypis trichas* (L.)	6	19	0/0	0/1	0/0	2/6, 12NT	2/6 (33)
Rose-breasted Grosbeak, *Pheucticus ludovicianus* (L.)	2	3	0/0	0/0	0/0	1/1, 2NT	1/1 (100)
Chestnut-sided Warbler, *Setophaga pensylvanica* (L.)	1	1	0/0	0/0	0/0	0/0, 1NT	0/0 (0)
Veery, *Catharus fuscescens* (Stephens)	8	26	0/1	0/2	1NT	6/19, 3NT	6/19 (32)
Song Sparrow, *Melospiza melodia* (Wilson)	1	3	0/0	0/0	0/0	0/0, 3NT	0/0 (0)
Gray Catbird, *Dumetella carolinensis* (L.)	1	1	0/0	0/0	0/0	0/1	0/1 (0)
Nashville Warbler, *Oreothlypsis ruficapilla* (Wilson)	1	1	0/0	0/0	0/0	0/1	0/1 (0)
Total: 8 bird species	23	68	0/1	0/3	1NT	14/33, 23NT	14/33 (42)

Bbss, *Borrelia burgdorferi* sensu stricto. Hlp, *Haemaphysalis leporispalustris*; Isc, *Ixodes scapularis*. NT, not tested. * Seven *I. scapularis* nymphs collected from an American Robin were pooled for testing, but were not included in the infection prevalence calculations for Bbsl.

**Table 2:** Results of sequence analysis of *Borrelia* species positive for *Borrelia burgdorferi* sensu lato and associated NCBI GenBank accession numbers detected in *Ixodes scapularis* nymphs collected from nesting songbirds at Montée Biggar, Québec, 2019.

Bird Species	Reference Strain	*flaB* Gene Sequence
American Robin *	CN19-71	MT039724
American Robin	CN19-75	MT039725
Common Yellowthroat	CN19-76B	MT039726
Rose-breasted Grosbeak	CN19-77	MT039727
Veery	CN19-109A	MT039728
American Robin †	CN19-112A-1	MT039729
American Robin †	CN19-112A-2	MT039730
American Robin †	CN19-112B-1	MT039731
American Robin †	CN19-112B-2	MT039732
Veery	CN19-113A	MT039733
Veery §	CN19-117A-2	MT039735
Veery §	CN19-117B-1	MT039736
Veery	CN19-118B	MT039737
Common Yellowthroat	CN19-121	MT093795

* pool of seven *I. scapularis* nymphs; †, same host bird; §, single avian host.

## References

[1] Nicholson W.A., Sonenshine D.E., Noden B.H., Mullen G.R., Durden L.A. (2019). Ticks (Ixodida). Medical and Veterinary Entomology.

[2] Scott J.D., Fernando K., Banerjee S.N., Durden L.A., Byrne S.K., Banerjee M., Mann R.B., Morshed M.G. (2001). Birds disperse ixodid (Acari: Ixodidae) and *Borrelia burgdorferi*-infected ticks in Canada. J. Med. Entomol..

[3] Ogden N.H., Lindsay L.R., Hanincová K., Barker I.K., Bigras-Poulin M., Charron D.F., Heagy A., Francis C.M., O’Callaghan C.J., Schwartz I. (2008). Role of migratory birds in introduction and range expansion of *I. scapularis* ticks and of *Borrelia burgdorferi* and *Anaplasma phagocytophilum* in Canada. Appl. Environ. Microbiol..

[4] Scott J.D., Lee M.-K., Fernando K., Durden L.A., Jorgensen D.R., Mak S., Morshed M.G. (2010). Detection of Lyme disease spirochete, *Borrelia burgdorferi* sensu lato, including three novel genotypes in ticks (Acari: Ixodidae) collected from songbirds (Passeriformes) across Canada. J. Vector Ecol..

[5] Scott J.D., Anderson J.F., Durden L.A. (2012). Widespread dispersal of *Borrelia burgdorferi*-infected ticks collected from songbirds across Canada. J. Parasitol..

[6] Scott J.D., Mahala G. (2015). Birds Widely Disperse Pathogen-Infected Ticks. Seabirds and Songbirds: Habitat Preferences, Conservation, Migratory Behavior.

[7] Scott J.D., Clark K.L., Foley J.E., Bierman B.C., Durden L.A. (2018). Far-reaching dispersal of *Borrelia burgdorferi* sensu lato-infected blacklegged ticks by migratory songbirds in Canada. Healthcare.

[8] Scott J.D., Clark K.L., Foley J.E., Anderson J.F., Bierman B.C., Durden L.A. (2018). Extensive distribution of the Lyme disease bacterium, *Borrelia burgdorferi* sensu lato, in multiple tick species parasitizing avian and mammalian hosts across Canada. Healthcare.

[9] DeLuca W.V., Woodworth B.K., Rimmer C.C., Marra P.P., Taylor P.D., McFarland K.P., Mackenzie S.A., Norris D.R. (2015). Transoceanic migration by a 12 g songbird. Biol. Lett..

[10] Hoogstraal H., Kaiser M.N. (1961). Ticks from European-Asiatic birds migrating through Egypt into Africa. Science.

[11] Scott J.D., Durden L.A. (2015). *Amblyomma dissimile* Koch (Acari: Ixodidae) parasitizes bird captured in Canada. Syst. Appl. Acarol..

[12] Morshed M.G., Scott J.D., Fernando K., Beati L., Mazerolle D.F., Geddes G., Durden L.A. (2005). Migratory songbirds disperse ticks across Canada, and first isolation of the Lyme disease spirochete, *Borrelia burgdorferi*, from the avian tick, *Ixodes auritulus*. J. Parasitol..

[13] Scott J.D., Durden L.A. (2015). First record of *Amblyomma rotundatum* tick (Acari: Ixodidae) parasitizing a bird collected in Canada. Syst. Appl. Acarol..

[14] Scott J.D., Durden L.A. (2015). New records of the Lyme disease bacterium in ticks collected from songbirds in central and eastern Canada. Int. J. Acarol..

[15] Scott J.D., Clark K.L., Durden L.A., Manord J.M., Smith M.L. (2016). First record of *Ixodes affinis* tick (Acari: Ixodidae) infected with *Borrelia burgdorferi* sensu lato collected from a migratory songbird in Canada. J. Bacteriol. Parasitol..

[16] Scott J.D., Durden L.A. (2015). Songbird-transported tick *Ixodes minor* (Ixodida: Ixodidae) discovered in Canada. Can. Entomol..

[17] Anderson J.F., Magnarelli L.A. (1984). Avian and mammalian hosts for spirochete-infected ticks and insects in a Lyme disease focus in Connecticut. Yale J. Biol. Med..

[18] Anderson J.F., Magnarelli L.A., Stafford K.C. (1990). Bird-feeding ticks transstadially transmit *Borrelia burgdorferi* that infect Syrian hamsters. J. Wildl. Dis..

[19] Scott J.D., Scott C.M., Anderson J.F. (2014). The establishment of a blacklegged tick population by migratory songbirds in Ontario, Canada. J. Veter Sci. Med..

[20] Burgdorfer W., Barbour A.G., Hayes S.F., Benach J.L., Grunwaldt E., Davis J.P. (1982). Lyme disease—A tick-borne spirochetosis?. Science.

[21] Baranton G., Postic D., Saint Girons I., Boerlin P., Piffaretti J.-C., Assous M., Grimont P.A.D. (1992). Delineation of *Borrelia burgdorferi* sensu stricto, *Borrelia garinii* sp. nov., and Group VS461 associated with Lyme borreliosis. Int. J. Syst. Bacteriol..

[22] Scott J.D., Clark K.L., Anderson J.F., Foley J.E., Young M.R., Durden L.A. (2017). Lyme disease bacterium, *Borrelia burgdorferi* sensu lato, detected in multiple tick species at Kenora, Ontario, Canada. J. Bacteriol. Parasitol..

[23] Scott J.D., Foley J.E. (2016). Detection of *Borrelia americana* in the avian coastal tick, *Ixodes auritulus* (Acari: Ixodidae), collected from a bird captured in Canada. Open J. Anim. Sci..

[24] Scott J.D., Clark K.L., Foley J.E., Anderson J.F., Durden L.A., Manord J.M., Smith M.L. (2017). Detection of *Borrelia* genomospecies 2 in *Ixodes spinipalpis* ticks collected from a rabbit in Canada. J. Parasitol..

[25] Sertour N., Cotté V., Garnier M., Malandrin L., Ferguel E., Choumet V. (2018). Infection kinetics and tropism of *Borrelia burgdorferi* sensu lato in mouse after natural (via ticks) or artificial (needle) infection depends on the bacterial strain. Front. Microbiol..

[26] Hynote E.D., Mervine P.C., Stricker R.B. (2012). Clinical evidence for rapid transmission of Lyme disease following a tickbite. Diagn. Microbiol. Infect. Dis..

[27] Cook M.J. (2015). Lyme borreliosis: A review of data on transmission time after tick attachment. Int. J. Gen. Med..

[28] Eisen R.J., Eisen L., Beard C.B. (2016). County-scale distribution of *Ixodes scapularis* and *Ixodes pacificus* (Acari: Ixodidae) in the continental United States. J. Med. Entomol..

[29] Clifford C.M., Anastos G., Elbl A. (1961). The larval ixodid ticks of the eastern United States. Misc. Publ. Entomol. Soc. Am..

[30] Keirans J.E., Hutcheson H.J., Durden L.A., Klompen J.S.H. (1996). *Ixodes* (*Ixodes*) *scapularis* (Acari: Ixodidae): Redescription of all active stages, distribution, hosts, geographical variation, and medical and veterinary importance. J. Med. Entomol..

[31] Durden L.A., Keirans J.E. (1996). Nymphs of the Genus Ixodes (Acari: Ixodidae) of the United States: Taxonomy, Identification Key, Distribution, Hosts, and Medical/Veterinary Importance. Monographs: Thomas Say Publications in Entomology.

[32] Cooley R.A. (1946). The Genera *Boophilus*, *Rhipicephalus*, and *Haemaphysalis* (Ixodidae) of the New World. National Institute of Health Bulletin No. 187.

[33] Barbour A.G., Maupin G.O., Teltow G.J., Carter C.J., Piesman J. (1996). Identification of an uncultivable *Borrelia* species in the hard tick *Amblyomma americanum*: Possible agent of a Lyme disease-like illness. J. Infect. Dis..

[34] Barbour A.G., Bunikis J., Travinsky B., Hoen A.G., Diuk-Wasser M.A., Fish D., Tsao J.I. (2009). Niche partitioning of *Borrelia burgdorferi* and *Borrelia miyamotoi* in the same tick vector and mammalian reservoir species. Am. J. Trop. Med. Hyg..

[35] Clark K., Hendricks A., Burge D. (2005). Molecular identification and analysis of *Borrelia burgdorferi* sensu lato in lizards in the southeastern United States. Appl. Environ. Microbiol..

[36] Thompson J.D., Gibson T.J., Plewniak F., Jeanmougin F., Higgins D.G. (1997). The ClustalX–Windows interface: Flexible strategies for multiple sequence alignment aided by quality analysis tools. Nucleic Acids Res..

[37] Altschul S.F., Gish W., Miller W., Myers E.W., Lipman D.J. (1990). Basic local alignment search tools. J. Mol. Biol..

[38] Edgar R.C. (2004). MUSCLE: Multiple sequence alignment with high accuracy and high throughput. Nucleic Acids Res..

[39] Mannelli A., Kitron U., Jones C.J., Slajchert T.L. (1994). Influence of season and habitat on *Ixodes scapularis* infestation on white-footed mice in northeastern Illinois. J. Parasitol..

[40] Peavey C.A., Lane R.S. (1995). Transmission of *Borrelia burgdorferi* by *Ixodes pacificus* nymphs and reservoir competence of deer mice (*Peromyscus maniculatus*) infected by tick-bite. J. Parasitol..

[41] McLean R.G., Ubico S.R., Cooksey L.M. (1993). Experimental infection of the eastern chipmunk (*Tamias striatus*) with the Lyme disease spirochete (*Borrelia burgdorferi*). J. Wildl. Dis..

[42] Rand P.W., Lacombe E.H., Smith R.P., Rich S.M., Kilpatrick C.A., Dragoni C.A., Caporale D. (1993). Competence of *Peromyscus maniculatus* (Rodentia: Cricetidae) as a reservoir host for *Borrelia burgdorferi* (Spirochaetales: Spirochaetaceae) in the wild. J. Med. Entomol..

[43] Anderson J.F., Johnson R.C., Magnarelli L.A., Hyde F.W. (1986). Involvement of birds in the epidemiology of the Lyme disease agent *Borrelia burgdorferi*. Infect Immun..

[44] Rand P.W., Lacombe E.H., Smith R.P., Ficker J. (1998). Participation of birds (Aves) in the emergence of Lyme disease in southern Maine. J. Med. Entomol..

[45] Stafford K.C., Bladen V.C., Magnarelli L.A. (1995). Ticks (Acari: Ixodidae) infesting wild birds (Aves) and white-footed mice in Lyme, CT. J. Med. Entomol..

[46] Richter D., Spielman A., Komar N., Matuschka F.-R. (2000). Competence of American Robins as reservoir hosts for Lyme disease spirochetes. Emerg. Infect. Dis..

[47] Banerjee S.N., Banerjee M., Fernando K., Dong M.Y., Smith J.A., Cook D. (1995). Isolation of *Borrelia burgdorferi*, the Lyme disease spirochete from rabbit ticks, *Haemaphysalis leporispalustris* from Alberta. J. Spir. Tick-Borne Dis..

[48] Scott J.D., Clark K.L., Coble N.M., Ballantyne T.R. (2019). Detection and transstadial passage of *Babesia* species and *Borrelia burgdorferi* sensu lato in ticks collected from avian and mammalian hosts in Canada. Healthcare.

[49] Banerjee S.N., Morshed M.G., Scott J.D. (2000). Epizootiology of the Lyme Disease Spirochete, Borrelia burgdorferi in Blacklegged Ticks, Ixodes scapularis and Small Mammals at Point Pelee National Park.

[50] Scott J.D., Fernando K., Durden L.A., Morshed M.G. (2004). Lyme disease spirochete, *Borrelia burgdorferi*, endemic in epicenter at Turkey Point, Ontario. J. Med. Entomol..

[51] Scott J.D., Lee M.-K., Fernando K., Jorgensen D.R., Durden L.A., Morshed M.G. (2008). Rapid introduction of Lyme disease spirochete, *Borrelia burgdorferi* sensu stricto, in *Ixodes scapularis* (Acari: Ixodidae) established at Turkey Point Provincial Park, Ontario, Canada. J. Vector Ecol..

[52] Morshed M.G., Scott J.D., Banerjee S.N., Fernando K., Mann R., Isaac-Renton J. (2000). First isolation of Lyme disease spirochete, *Borrelia burgdorferi* from blacklegged tick, *Ixodes scapularis*, collected at Rondeau Provincial Park, Ontario. Can. Commun. Dis. Rep..

[53] Morshed M.G., Scott J.D., Fernando K., Mann R.B., Durden L.A. (2003). Lyme disease spirochete, *Borrelia burgdorferi* endemic at epicenter in Rondeau Provincial Park, Ontario. J. Med. Entomol..

[54] Scott J.D., Anderson J.F., Durden L.A., Smith M.L., Manord J.M., Clark K.L. (2016). Prevalence of the Lyme disease spirochete, *Borrelia burgdorferi*, in blacklegged ticks, *Ixodes scapularis* at Hamilton-Wentworth, Ontario. Int. J. Med. Sci..

[55] Scott J.D., Foley J.E., Anderson J.F., Clark K.L., Durden L.A. (2017). Detection of Lyme disease bacterium, *Borrelia burgdorferi* sensu lato, in blacklegged ticks collected in the Grand River Valley, Ontario, Canada. Int. J. Med. Sci..

[56] Banerjee S., Banerjee M., Scott J., Lankester M., Kubinec J. (1996). Isolation of *Borrelia burgdorferi*−Thunder Bay District, Ontario. Can. Commun. Dis. Rep..

[57] Banerjee S.N., Christensen C.I., Scott J.D. (1995). Isolation of *Borrelia burgdorferi* on mainland Ontario. Can. Commun. Dis. Rep..

[58] Scott J.D., Foley J.E., Clark K.L., Anderson J.F., Durden L.A., Manord J.M., Smith M.L. (2016). Established population of blacklegged ticks with high infection prevalence for the Lyme disease bacterium, *Borrelia burgdorferi* sensu lato, on Corkscrew Island, Kenora District, Ontario. Int. J. Med. Sci..

[59] Cameron D.J., Johnson L.B., Maloney E.L. (2014). Evidence assessments and guideline recommendations in Lyme disease: The clinical management of known tick bites, erythema migrans rashes and persistent disease. Expert Rev. Anti-Infect. Ther..

[60] Shor S., Green C., Szantyr B., Phillips S., Liegner K.L., Burrascano J., Bransfield R., Maloney E.L. (2019). Chronic Lyme disease: An evidence-based definition by the ILADS Working Group. Antibiotics.

[61] Johnson L., Shapiro M., Mankoff J. (2018). Removing the mask of average treatment effects in chronic Lyme disease research using Big Data and subgroup analysis. Healthcare.

[62] Berger B.W. (1989). Dermatologic manifestations of Lyme disease. Rev. Infect. Dis..

[63] Stonehouse A., Studdiford J.S., Henry C.A. (2010). An update on the diagnosis and treatment of early Lyme disease: “focusing on the bull’s eye, you may miss the mark”. J. Emerg. Med..

[64] Johnson L., Mankoff J., Stricker R.B. (2014). Severity of chronic Lyme disease compared to other chronic conditions: A quality of life survey. PeerJ.

[65] Sapi E., MacDonald A. (2008). Biofilms of *Borrelia burgdorferi* in chronic cutaneous borreliosis. Am. J. Clin. Pathol..

[66] MacDonald A.B. (1988). Concurrent neocortical borreliosis and Alzheimer’s disease: Demonstration of a spirochetal cyst form. Ann. NY Acad. Sci..

[67] Meriläinen L., Herranen A., Schwarzbach A., Gilbert L. (2015). Morphological and biochemical features of *Borrelia burgdorferi* pleomorphic forms. Microbiology.

[68] Oksi J., Kalimo H., Marttila R.J., Marjamäki M., Sonninen P., Nikoskelainen J., Viljanen M.K. (1996). Inflammatory brain changes in Lyme borreliosis: A report on three patients and review of literature. Brain.

[69] MacDonald A.B. (2007). Alzheimer’s neuroborreliosis and trans-synaptic spread of infection and neurofibrillary tangles derived from intraneuronal spirochetes. Med. Hypotheses.

[70] Miklossy J. (2011). Alzheimer’s disease—A neurospirochetosis. Analysis of the evidence following Koch’s and Hill’s criteria. J. Neuroinflamm..

[71] Sapi E., Kasliwala R.S., Ismail H., Torres J.P., Oldakowski M., Markland S., Gaur G., Melillo A., Eisendle K., Liegner K.B. (2019). The long-term persistence of *Borrelia burgdorferi* antigens and DNA in the tissues of a patient with Lyme disease. Antibiotics.

[72] Oksi J., Mertsola J., Reunanen M., Marjamäki M., Viljanen M.K. (1994). Subacute multiple-site osteomyelitis caused by *Borrelia burgdorferi*. Clin. Infect. Dis..

[73] Preac-Mursic V., Pfister H.W., Spiegel H., Burk R., Wilske B., Reinhardt S., Böhmer R. (1993). First isolation of *Borrelia burgdorferi* from an iris biopsy. J. Clin. Neuroophthalmol..

[74] Häupl T., Hahn G., Rittig M., Krause A., Schoerner C., Schönherr U., Kalden J.R., Burmester G.R. (1993). Persistence of *Borrelia burgdorferi* in ligamentous tissue from a patient with chronic Lyme borreliosis. Arthr. Rheum..

[75] Müller M.E. (2012). Damage of collagen and elastic fibres by *Borrelia burgdorferi*―Known and new clinical histopathogical aspects. Open Neurol. J..

[76] Preac-Mursic V., Marget W., Busch U., Pleterski Rigler D., Hagl S. (1996). Kill kinetics of *Borrelia burgdorferi* and bacterial findings in relation to the treatment of Lyme borreliosis. Infection.

[77] Frey M., Jaulhac B., Piemont Y., Marcellin L., Boohs P.M., Vautravers P., Jesel M., Kuntz J.L., Monteil H., Sibilia J. (1998). Detection of *Borrelia burgdorferi* DNA in muscle of patients with chronic myalgia related to Lyme disease. Am. J. Med..

[78] Girschick H.J., Huppertz H.I., Rüssmann H., Krenn V., Karch H. (1996). Intracellular persistence of *Borrelia burgdorferi* in human synovial cells. Rheumatol. Int..

[79] Garcia-Monco J.C., Villar F.F., Alen J.C., Benach J.L. (1990). *Borrelia burgdorferi* in the central nervous system: Experimental and clinical evidence for early invasion. J. Infect. Dis..

[80] Luft B.J., Steinman C.R., Neimark H.C. (1992). Invasion of the central nervous system by *Borrelia burgdorferi* in acute disseminated infection. JAMA.

[81] Livengood J.A., Gilmore R.D. (2006). Invasion of human neuronal and glial cells by an infectious strain of *Borrelia burgdorferi*. Microbes Infect..

[82] Ramesh G., Borda J.T., Dufour J., Kaushal D., Ramamoorthy R., Lackner A.A., Philipp M.T. (2008). Interaction of the Lyme disease spirochete *Borrelia burgdorferi* with brain parenchyma elicits inflammatory mediators from glial cells as well as glial and neuronal apoptosis. Am. J. Pathol..

[83] Ramesh G., Santana-Gould L., Inglis F.M., England J.D., Philipp M.T. (2013). The Lyme disease spirochete *Borrelia burgdorferi* induces inflammation and apoptosis in cells from dorsal root ganglia. J. Neuroinflamm..

[84] Klempner M.S., Noring R., Rogers R.A. (1993). Invasion of human skin fibroblasts by the Lyme disease spirochete, *Borrelia burgdorferi*. J. Infect. Dis..

[85] Stricker R.B. (2007). Counterpoint: Long-Term antibiotic therapy improves persistent symptoms associated with Lyme disease. Clin. Infect. Dis..

[86] Hodzic E., Feng S., Holden K., Freet K.J., Barthold S.W. (2008). Persistence of *Borrelia burgdorferi* following antibiotic treatment in mice. Antimicrob. Agents Chemother..

[87] Embers M.E., Barthold S.W., Borda J.T., Bowers L., Doyle L., Hodzic E., Jacobs M.B., Hasenkampf N.R., Martin D.S., Narasimhan S. (2012). Persistence of *Borrelia burgdorferi* in rhesus macaques following antibiotic treatment of disseminated infection. PLoS ONE.

[88] Liegner K.B., Duray P., Agricola M., Rosenkilde C., Yannuzzi L.A., Ziska M., Tilton R.C., Hulinska D., Hubbard J., Fallon B.A. (1997). Lyme disease and the clinical spectrum of antibiotic responsive chronic meningoencephalomyelitides. J. Spir. Tick-Borne Dis..

[89] Middelveen M.J., Sapi E., Burke J., Filush K.R., Franco A., Fesler M.C., Stricker R.B. (2018). Persistent *Borrelia* infection in patients with ongoing symptoms of Lyme disease. Healthcare.

[90] Miklossy J., Kasas S., Zurn A.D., McCall S., Yu S., McGeer P.L. (2008). Persisting atypical and cystic forms of *Borrelia burgdorferi* and local inflammation in Lyme neuroborreiosis. J. Neuroinflamm..

[91] Coyle P.K., Schutzer S.E., Deng Z., Krupp L.B., Belman A.L., Benach J.L., Luft B.J. (1995). Detection of *Borrelia burgdorferi*-specific antigen in antibody-negative cerebrospinal fluid in neurologic Lyme disease. Neurology.

[92] Mikkilä H.O., Seppälä I.J., Viljanen M.K., Peltomaa M.P., Karma A. (2000). The expanding clinical spectrum of ocular Lyme borreliosis. Ophthalmology.

[93] Preac-Mursic V., Weber K., Pfister H.W., Wilske B., Gross B., Baumann A., Prokop J. (1989). Survival of *Borrelia burgdorferi* in antibiotically treated patients with Lyme borreliosis. Infection.

[94] Battafarano D.F., Combs J.A., Enzenauer R.J., Fitzpatrick J.E. (1993). Chronic septic arthritis caused by *Borrelia burgdorferi*. Clin. Orthop. Relat. Res..

[95] Chancellor M.B., McGinnis D.E., Shenot P.J., Kiilholma P., Hirsch I.H. (1993). Urinary dysfunction in Lyme disease. J. Urol..

[96] Lawrence C., Lipton R., Lowy R., Coyle P.K. (1995). Seronegative chronic relapsing neuroborreliosis. Eur. Neurol..

[97] Hudson B.J., Stewart M., Lennox V.A., Fukunaga M., Yabuki M., Macorison H., Kitchener-Smith J. (1998). Culture-positive Lyme borreliosis. Med. J. Aust..

[98] Oksi J., Nikoskelainen J., Hiekkanen H., Lauhio A., Peltomaa M., Pitkäranta A., Nyman D., Granlund H., Carlsson S.A., Seppälä I. (2007). Duration of antibiotic treatment in disseminated Lyme borreliosis: A double-blind, randomized, placebo-controlled, multicenter clinical study. Eur. J. Clin. Microbiol. Infect. Dis..

[99] Fraser D.D., Kong L.I., Miller F.W. (1992). Molecular detection of persistent *Borrelia burgdorferi* in a man with dermatomyositis. Clin. Exp. Rheumatol..

[100] Stricker R.B., Fesler M.C. (2018). Chronic Lyme disease: A working case definition. Am. J. Infect. Dis..

[101] Davidsson M. (2018). The financial implications of a well-hidden and ignored chronic Lyme disease pandemic. Healthcare.

[102] Fallon B.A., Pavlicova M., Coffino S.W., Brenner C. (2014). A comparison of Lyme disease serologic test results from four laboratories in patients with persistent symptoms after antibiotic treatment. Clin. Infect. Dis..

[103] Stricker R.B., Johnson L. (2010). Lyme disease diagnosis and treatment: Lessions from the AIDS epidemic. Minerva Med..

[104] Schubert H.D., Greenebaum E. (1994). Cytologically proven seronegative Lyme choroiditis and vitriitis. Retina.

[105] Schutzer S.E., Coyle P.K., Belman A.L., Golightly M.G., Drulle J. (1990). Sequestration of antibody to *Borrelia burgdorferi* in immune complexes in seronegative Lyme disease. Lancet.

[106] Schmidli J., Hunziker T., Moesli P., Schaad U.B. (1988). Cultivation of *Borrelia burgdorferi* from joint fluid three months after treatment of facial palsy due to Lyme borreliosis. J. Infect. Dis..

[107] Berndtson K. (2013). Review of evidence from immune evasion and persistent infection in Lyme disease. Int. J. Gen. Med..

[108] Bransfield R.C. (2017). Suicide and Lyme and associated diseases. Neuropsychiatr. Dis. Treat..

[109] Bransfield R.C. (2018). Aggressiveness, violence, homocidality, homicide, and Lyme disease. Neuropsychiatr. Dis. Treat..

[110] Bransfield R.C., Cook M.J., Bransfield D.R. (2019). Proposed Lyme disease guidelines and psychiatric illnesses. Healthcare.

[111] Cassarino D.S., Quezado M.M., Ghatak N.R., Duray P.H. (2003). Lyme-associated parkinsonism: A neuropathologic case study and review of the literature. Arch. Pathol. Lab. Med..

[112] Bertrand E., Szpak G.M., Pilkowska E., Habib N., Lipczyńska-Lojkowska W., Rudnicka A., Tylewska-Wierzbanowska S., Kulczycki J. (1999). Central nervous system infection caused by *Borrelia burgdorferi*. Clinico-pathological correlation of three post-mortem cases. Folia Neuropathol..

[113] Fesler M.C., Middelveen M.J., Burke J.M., Stricker R.B. (2019). Erosive vulvovaginitis associated with *Borrelia burgdorferi* infection. J. Investig. Med. High Impact Case Rep..

[114] Lavoie P.E., Lattner B.P., Duray P.H., Barbour A.G., Johnson P.C. (1987). Culture positive seronegative transplacental Lyme borreliosis infant mortality. Arthr. Rheum..

[115] MacDonald A.B., Johnson R.C. (1989). Gestational Lyme Borreliosis: Implication for the Fetus. Rheumatic Disease Clinics of North America.

[116] Trevisan G., Stinco G., Cinco M. (1997). Neonatal skin lesions due to spirochetal infection: A case of congenital Lyme borreliosis?. Int. J. Dermatol..

[117] Horowitz R.I. Lyme Disease and Pregnancy: Implication of Chronic Infection, PCR Testing, and Prenatal Treatment. Proceedings of the 16th International Scientific Conference on Lyme Disease & Other Tick-Borne Disorders.

[118] MacDonald A.B., Benach J.L., Burgdorfer W. (1987). Stillbirth following maternal Lyme disease. NY State J. Med..

[119] Gardner T., Remington J.S., Klein J.O. (2001). Lyme Disease. Infectious Diseases of the Fetus and Newborn Infant.

